# Supplementary material for: Backstepping Mechanism of Kinesin-1
Source: Biophys J. 2020 Oct 6;119(10):1984–94. doi: 10.1016/j.bpj.2020.09.034 (PMC7732724; doi:10.1016/j.bpj.2020.09.034)
Supplement: Document S1. Figs. S1 and S2 [file mmc1.pdf]

Biophysical Journal, Volume *119*

Supplemental Information

Backstepping Mechanism of Kinesin-1

Algirdas Toleikis, Nicholas J. Carter, and Robert A. Cross

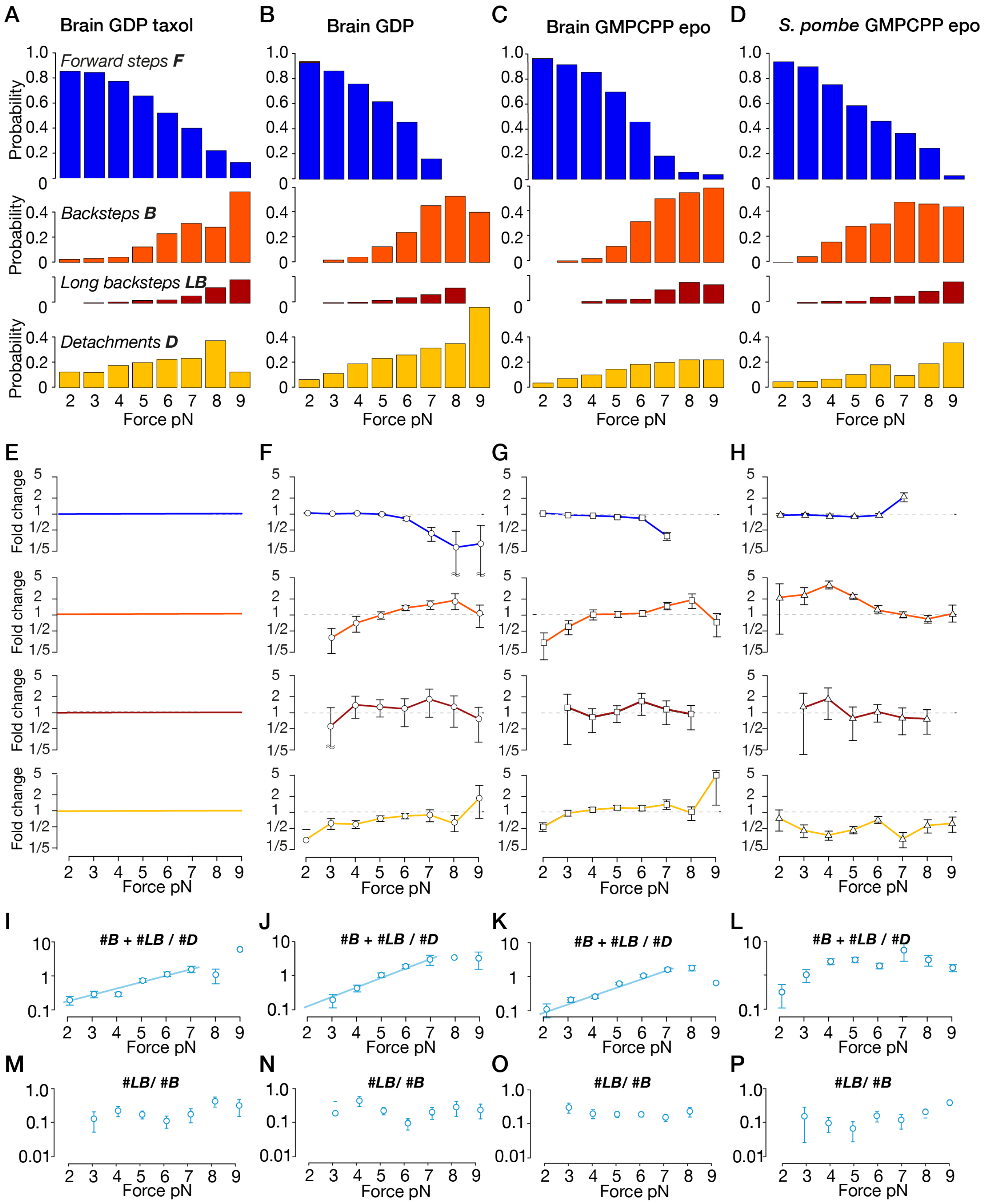

Supplementary Fig. 1. Event counts versus MT lattice type. Further analysis of relative event counts versus load. (A-D) Relative event counts for different lattice types; replots of the data shown in Fig.3A-D, facilitating comparison of the effects of changing MT lattice type. (E-H) fold change in these same event counts, compared to GDP taxol MTs. (F) and (G) are relative to brain GDP taxol MTs; (H) is relative to brain GMPCPP epothilone MTs, so that only the species, and not the species and the drug, varies. (I-L) ratio of all backstep events to detachments versus load, for different lattice types. (M-P) ratio of long backsteps to 8 nm backsteps versus load, for different lattice types.

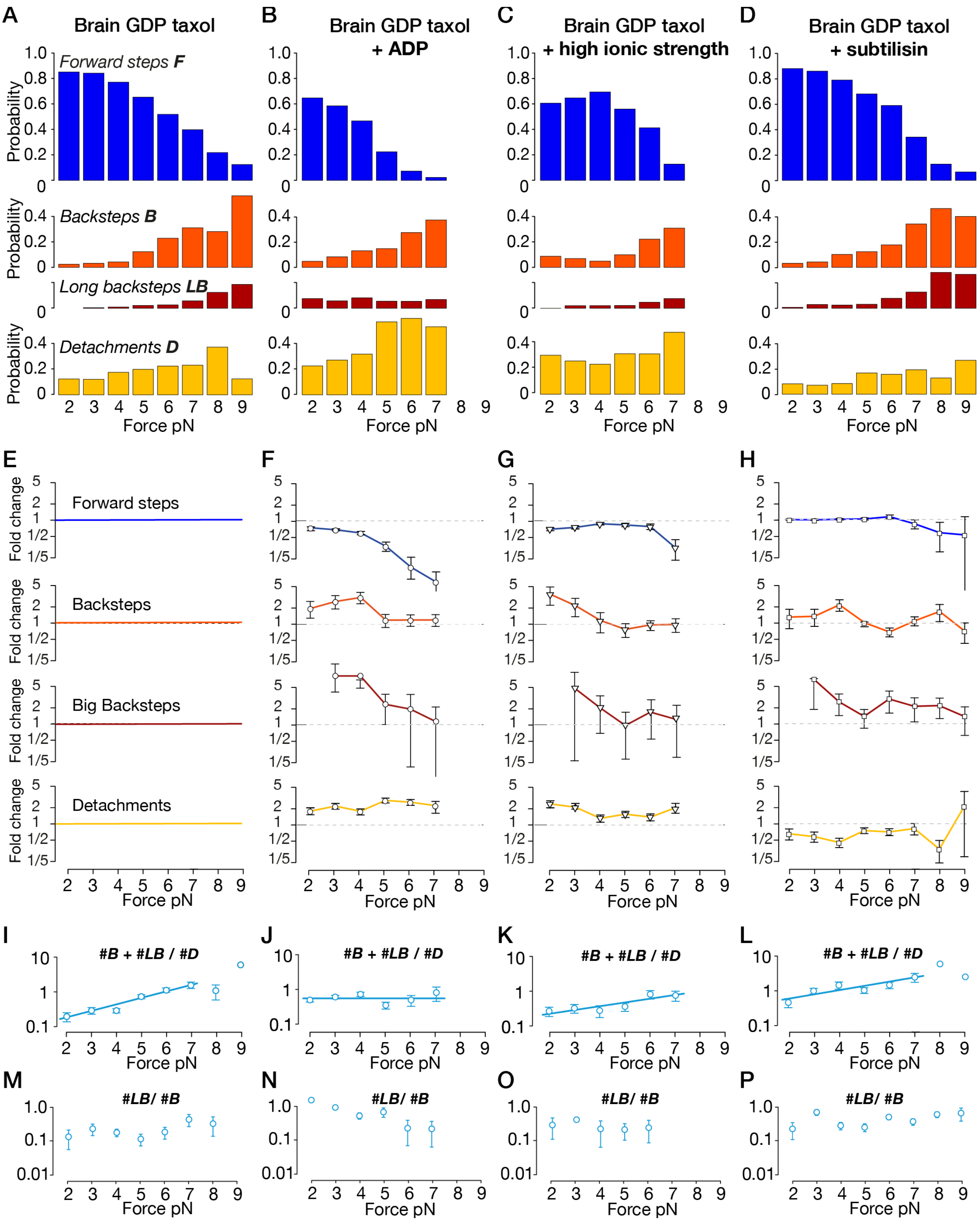

Supplementary Fig. 2. Event counts under varying conditions. Further analysis of relative event counts versus load. (A-D) Relative event counts for different conditions, replots of the data shown in Fig.4A-D, facilitating comparison of the effects of changing MT lattice type. (E-H) fold change in these same event counts, compared to GDP taxol MTs. ADP suppresses forward stepping, ehnaces backstepping and detachment, and adds a population of long backteps at low load that reduces with increasing load. (I-L) ratio of all backstep events to detachments versus load, for different conditions. (M-P) ratio of long backsteps to 8nm backsteps versus load, for different lattice types.
